# Undertaking a randomised controlled trial in the police setting: methodological and practical challenges

**DOI:** 10.1186/s13063-017-2369-6

**Published:** 2017-12-22

**Authors:** Arabella Scantlebury, Catriona McDaid, Alison Booth, Caroline Fairhurst, Adwoa Parker, Rebecca Payne, Helen Reed, William J. Scott, David Torgerson, Catherine Hewitt

**Affiliations:** 1 York Trials Unit, Department of Health Sciences, University of York, York, YO10 5DD UK; 2 North Yorkshire Police, Alverton Court, Northallerton, North Yorkshire DL6 1BF UK

**Keywords:** Randomised controlled trials, Police, Pragmatic

## Abstract

**Background:**

There has been an increased drive towards Evidence Based Policing in recent years. Unlike in other public sector services, such as health and education, randomised controlled trials in the police setting are relatively rare. This paper discusses some of the methodological and practical challenges of conducting a randomised controlled trial in the police setting in the UK, based on our experience of the Connect trial. This pragmatic, cluster-randomised controlled trial investigated the effectiveness of a face-to-face training intervention for frontline officers in comparison to routine training. The primary outcome was the number of incidents which resulted in a police response reported to North Yorkshire Police control room in a 1-month period up to 6 months after delivery of training.

**Main text:**

The methodological and practical challenges that we experienced whilst conducting the Connect trial are discussed under six headings: establishing the unit of randomisation; population of interest and sample size; co-production of evidence; time frame; outcomes; and organisational issues.

**Conclusion:**

Recommendations on the conduct of future randomised controlled trials in the police setting are made. To understand the context in which research is undertaken, collaboration between police and academia is needed and police officers should be embedded within trial management groups. Engagement with police data analysts to understand what data is available and facilitate obtaining trial data is also recommended. Police forces may wish to review their IT systems and recording practices. Pragmatic trials are encouraged and time frames need to allow for trial set-up and obtaining relevant ethical approvals.

**Trial registration:**

ISRCTN Registry, ID: ISRCTN11685602. Retrospectively registered on 13 May 2016.

## Background

There has been an increased global drive towards Evidence Based Policing (EBP) in recent years, as demonstrated by the creation of a number of Societies of Evidence Based Policing in England [1], Australia and New Zealand [2], the USA [3] and Canada [4]. The What Works Centre for Crime Reduction Toolkit, and a network of What Works Centres, has also been created to provide easy access to evidence to inform public spending and policy decisions [5].

In the UK, political interest in EBP is rising following the Prime Minister, Theresa May’s announcement in her role as Home Secretary that policing and crime reduction should have ‘the same relentless focus on evidence as our medical and legal professions – where knowledge and research are the foundation of professional practice’ [6]. The Police Knowledge Fund has also increased the UK Government’s financial commitment to EBP, by making £10million available to support the development of sustainable education and research collaborations between the police and academia in England and Wales [7].

Given the pressures on financing public services and the aim of EBP, which is to ensure that police decision-making is informed by the best available evidence, there is a need to make sure that we introduce interventions that are likely to be of benefit. Randomised controlled trials (RCTs) are often considered to be the ‘gold standard’ method of determining effectiveness [8]. However, there have been relatively few RCTs in the UK police service or elsewhere, especially when compared with other public services such as health or education [9]. There are examples of initiatives being implemented into police forces, without any robust evidence of their effectiveness. For example, the Crisis Intervention Team (CIT) model, a police-based response, which aims to improve how officers respond to situations involving individuals with mental health problems, is being implemented in police forces across the USA [10] despite their being no high-quality evidence of the CIT’s effectiveness [11].

In this paper we discuss some of the methodological and practical challenges of conducting an RCT in the police setting that we identified based on our experience of the Connect trial (ISRCTN registry, trial ID: 11685602). The Connect trial is part of the Co-Production of Policing Evidence, Research and Training: focus mental health (Connect) project, which was funded by the College of Policing (CoP), the Higher Education Funding Council for England and the Home Office. The Connect trial was conducted between September 2015 and March 2017 and investigated the effectiveness of a face-to-face mental health training intervention, delivered by mental health professionals to frontline officers, in comparison with routine training. The purpose of the training was to enhance officers’ ability to effectively identify individuals with mental ill-health and manage incidents with a mental health component. In doing so, the training aimed to reduce the likelihood of such individuals being involved in future incidents and reduce demand on police resources. The pre-specified primary outcome was the number of incidents reported to the police force’s control room which resulted in a police response. We acknowledge that assuming that the mental health training intervention will affect the number of incidents requiring a police response reported to the police control room is an indirect measure of effect and may mean that any beneficial effect could be diluted and/or take a while to observe. However, there is some evidence to suggest that dealing with reported incidents involving people with mental ill-health is a significant strain on police resources [12, 13]. If frontline officers received training in how to effectively manage such individuals, it was hoped that this would reduce the likelihood of these individuals being involved in future incidents, thereby reducing the number of incidents being reported to the police.

**Fig. 1:**
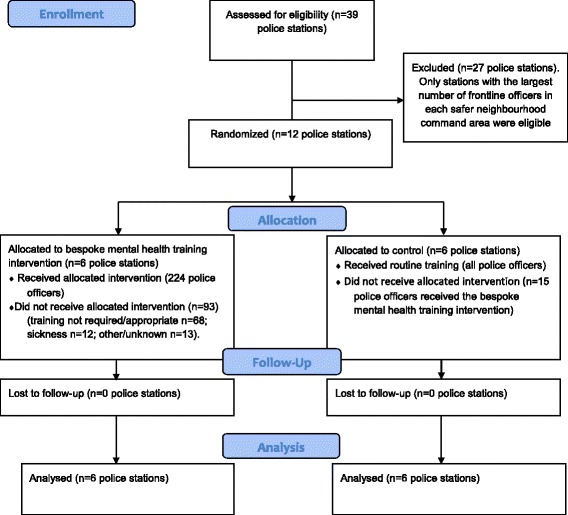
Consolidated Standards of Reporting Trials (CONSORT) 2010 flow diagram

Further details of the Connect trial are provided in Table 1 and Fig. 1, and the study has been published elsewhere [14]. Additional details of the Connect project, which includes a series of systematic reviews on mental health and policing, research methods training for police officers, work to understand current policing practices and their relationships with other agencies in the delivery of mental health services and an evaluation of the overall Connect project, can be found on the project’s website [15].

**Table 1:** The Connect trial

Objectives: To evaluate the effectiveness of a mental health training package for frontline officers relative to routine training
Design: A pragmatic, two-armed, cluster-randomised controlled trial, in a police force in the North of England. Twelve police stations were randomised, to receive the mental health training package (*n* = 6) or routine training (*n* = 6). Training for police officers is mandatory and so following approval from the police force, participation in the training was compulsory for eligible frontline officers reporting to stations that were allocated to receive the intervention. Three hundred and sixty officers were put forward for training, of whom 249 received the intervention.
Intervention: In addition to routine training, officers in the intervention group received a 1-day specialised mental health training package, delivered by mental health professionals. The training aimed to improve officers’ understanding of, and ability to: identify mental vulnerability; record relevant information using available systems; respond using appropriate internal and external resources; refer vulnerable people into services to provide longer-term assistance; and review incidents to make sure that risks have been effectively managed
Control: Officers in the control group were not informed of their allocation and did not receive any additional training outside of mandatory routine mental health training provided to all North Yorkshire officers (NYP). Mandatory routine mental health training for all NYP police officers includes: basic mental health law; specific NYP procedures around mental health and responding to incidents involving individuals with mental health problems; and a separate 2-3 hour online basic mental health training package.
Blinding: Due to the nature of the intervention, it was not feasible to blind police stations or individual police officers to the group they were allocated to; however, stations and officers allocated to the control group were not explicitly informed of their allocation
Outcome measures: The primary outcome was the number of incidents which resulted in a police response reported to the NYP control room over a 1-month period, 6 months after delivering training. Secondary outcomes included: likelihood of incidents having Section 136 of the Mental Health Act applied; likelihood of incidents having a mental health tag applied; and number of individuals with a mental health warning marker involved in any incident.
Trial status: Completed

## Main text

Here, we discuss the six main challenges that we faced whilst undertaking a pragmatic, cluster-RCT.

### Challenge 1: Establishing the unit of randomisation

To determine the most appropriate unit of randomisation and establish the risk of contamination based on how the units were defined, we needed to understand the police force’s organisational and geographical structure. We determined that individual randomisation to the training intervention was not appropriate because police officers often work in pairs or groups; therefore, there was a risk that officers in the control arm could have been exposed to the intervention, through interaction with colleagues randomised to the intervention arm. To minimise the risk of contamination, it was decided that cluster randomisation should be used, whereby a group of officers are randomised to either the intervention or control group as a single unit. The police force in this area currently operate across six geographical boundaries known as Safer Neighbourhood Command (SNC) areas, with each SNC containing varying numbers of police stations and officers. One option was to randomise at the level of the SNC, but six is considered an insufficient number of clusters for an RCT [16]; therefore, we decided that police stations were the most appropriate cluster. However, there remained a risk of contamination between stations as there is some movement of police officers between police stations; particularly among smaller stations where officers are often required to cover staff shortages and major events. We therefore included only the two largest stations within each of the six geographical areas that the police force operated across in order to minimise contamination between control and intervention stations. This decision was made with consideration to the sample size also, as discussed below.

### Challenge 2: Population of interest and sample size

Commonly, sample sizes in RCTs are determined by calculating the number of units of analysis required to be able to detect a meaningful difference in the outcome of interest [17]. However, there was limited data on which to base such a calculation in the current academic literature around incidents reported to the police, and what a meaningful reduction in incidents would be. Therefore, we took a more pragmatic approach to determine our sample size based on the minimum recommended number of clusters for a cluster-RCT, the type of police role that the police representatives on the team thought would benefit from the intervention, and the number of officers it was possible to train given the time and resource constraints. At least four clusters per arm are recommended for a cluster-RCT [16] so our sample of 12 police stations met this, and it was deemed feasible to train eligible officers from half this number of stations within the time frame. In order to establish this, the decision had to be made that ‘frontline’ officers only (i.e. those most likely to come into contact with members of the public/attend reported incidents) would be eligible for the trial since they were most likely to benefit from the messages delivered in the training intervention. This raised the issue of how to define a ‘frontline officer’. For example, should specialist officers such as those working in the firearms or dog-handling units be considered frontline officers? Resolving such issues benefitted greatly from the co-production aspect of the Connect project, which meant close working with police service representatives.

### Challenge 3: Understanding the policing context

A collaborative approach to designing, planning and implementing the trial was crucial to the successful delivery of the Connect trial. At the outset of the study we set up a Trial Management Group which included members of the trials unit undertaking the evaluation (statisticians, methodologists), the team developing the training intervention, and representatives from the police service. The multi-disciplinary nature of this group was essential to developing solutions to the various contextual issues that were well-informed in terms of what was practical and relevant for the police stakeholders; as well as being as methodologically robust as possible. The team met on a monthly basis, to discuss trial design, trial documentation and publications, interpretation of trial results and to oversee and input into monitoring and progress of the trial.

Given the implications that contextual information has for research design, we suggest that police officers are embedded within research teams and are involved in the design, delivery and evaluation of research relating to the police service. To achieve this, a similar philosophy is needed to that which was adopted for Patient and Public Involvement in clinical research [18, 19], to ensure that the co-production of evidence and embedding of police officers within research teams becomes the norm. It is our experience that a model where police officers are working in partnership with the research team, such as a co-production model, will not only strengthen research design and delivery, but may also raise police officers’ awareness of research procedures and create further opportunities for high-quality research to inform EBP.

### Challenge 4: Time frame and trial set-up

Another challenge was the time frame for the Connect trial. We had 18 months to design and conduct the trial and report the findings, which had a number of implications for trial design. It meant that the outcomes we could gather were limited as we did not have time to secure the necessary permissions to use patient data, i.e. to prospectively collect outcome data from individuals with mental ill-health involved in incidents attended by officers in the trial stations. This would have required obtaining ethical approval from the University and the Health Research Authority which can be a lengthy process. We therefore specified our outcomes in relation to data that is routinely collected by the police force, which was efficient, but limiting. The mental health training intervention also needed to be designed and implemented in sufficient time to allow enough time for a 6-month follow-up period, as this was considered the minimum time to observe any effect of the intervention. The timing of intervention delivery required close liaison with the police force’s training department. Police training has to be scheduled a minimum of 3 months in advance to ensure that adequate cover can be put in place and clashes with other police activities avoided. Attendance was also constrained by the number of officers that could be released from duties at one time. For example, it is not possible for a large number of police officers from a station to be released for training on the same day.

### Challenge 5: Outcomes

One of the greatest challenges that we faced during the design phase was identifying trial outcomes that were both meaningful and achievable. For example, we wanted to capture the service user perspective and would normally select a primary outcome that would reflect the impact on the people that the intervention is intended to benefit. In this case this was people with mental health issues coming into contact with the police. However, this was not possible given the timeframe so we were reliant on routinely collected police data. At the outset of the project the local police force identified five key areas that were of concern to them. These were: how frontline officers: (1) *identify* mental vulnerability, (2) *record* relevant information using available systems, (3) *respond* using appropriate internal and external resources, (4) *refer* vulnerable people into services to provide longer-term assistance and (5) *review* incidents to make sure that risks have been effectively managed. Whilst this gave us key areas to focus our outcomes on, we encountered significant issues in mapping these to routinely collected data. For example, for some of the priority areas, such as referral of individuals with mental health problems to other services, data was not recorded.

The majority of issues we encountered regarding outcome data related to the complex processes for routine recording of mental health incidents. The police routinely collect data from incidents, calls and contacts with members of the public, which are stored on a number of different IT systems. These systems all have slightly different purposes and capabilities, are accessed by police officers with differing roles and are not necessarily integrated with one another. A significant amount of work was, therefore, needed in order for us to understand for each of the police force’s five priority areas: what information was collected, where it was stored, which officers could access/edit information on each system and how we could access and link this data. One example of this is repeat callers. We originally aimed to address the police force’s rising concerns at the number of repeat calls that they receive from individuals with mental health problems, by considering the number of ‘frequent callers’ as a secondary outcome. However, data for this could not be obtained. All calls made to the force control room are ‘logged’ initially on the police’s telephone system, ‘Aspire’. Aspire identifies a telephone number and if the number has an associated contact record, the record appears so that force control room staff are able to see a caller’s identity. Each time an individual makes a call, this is logged on Aspire. However, the system does not create individual incident logs for each call. Additionally, Aspire data can only be extracted manually and there is no mechanism in the system for searching for all those who call on a frequent basis. An estimate of frequent callers can, therefore, only be determined by using the ‘Niche’ system. However, as each call is not logged individually on Niche this does not result in accurate data on the frequency of calls per caller.

One way to overcome challenges associated with identifying outcomes and obtaining routinely collected data in the police setting is to have engagement from police data analysts. In the Connect trial, a dedicated police data analyst extracted data from the police force’s IT systems and helped us to understand what data was available and how accurate it was likely to be.

The amount of routinely collected data recorded by police forces and the potential for using ‘Big Data’ is one of the most exciting aspects of EBP. However, for this potential to be utilised, when developing and refining their systems for routine data collection and prioritise what data is collected, police forces may wish to consider ways to maximise their ‘readiness for research’. Ideally, police systems should be designed with operational *and* research purposes in mind as currently data is stored on a number of different systems, which are not integrated and for which there are concerns about the accuracy and completeness of data entry. There may be lessons to learn from healthcare, where large data sets (e.g. Hospital Episode Statistics) are regularly used to facilitate high-quality research in the National Health Service (NHS).

### Challenge 6: Organisational issues

As with all pragmatic trials, shifting landscapes were a key challenge faced during the Connect trial. During our 18-month trial period, a number of initiatives were introduced by the police to try to assist their officers in dealing with the large number of incidents involving individuals with mental health problems. For example, at the start of the trial street triage had just been implemented across a number of stations and mental health triage nurses were introduced in the police force’s control room. Whilst we could ensure that intervention and control groups were balanced as to whether street triage was in operation (by including it as a factor in the randomisation using minimisation), other initiatives were more challenging to resolve.

During the planning of the Connect trial we identified a number of issues pertaining to how mental health incidents were recorded, namely that frontline officers were either not able, or did not have the awareness of how, to record information on incidents involving individuals with mental health problems. Understandably, the police force wanted to resolve this issue quickly and so proposed a pilot that would provide training to frontline officers at our largest intervention station on the recording of mental health incidents. This was a positive outcome of the co-production model used as we had raised the police force’s awareness of recording issues. However, two of our trial outcomes were around the recording of mental health incidents. Changing the awareness of recording practices for officers in one of the intervention group stations had the potential to dilute the effect of the intervention and potentially undermine the credibility of the trial. Whilst it was not possible to prevent the pilot, through discussion with the police force we were able to modify the mental health training intervention to include content on recording of mental health incidents. Ensuring that police officers are embedded within trial teams and attend regular trial meetings provides the opportunity for any new and/or proposed initiatives to be discussed and enables any necessary adjustments to trial design or intervention delivery to be made in a timely fashion.

As well as having practical implications for research, the shifting landscape of policing raises a broader ethical issue for EBP; namely, whether it is practical for the police to delay resolving issues until after a research project has finished, or if forces should continue to implement initiatives and risk undermining expensive research projects, which aim to provide evidence on which decisions should be based. There is a challenge here for academics as well and the need to be aware of the pace of policing policy change and the importance of conducting research in a timely fashion. This may involve the development of new methodologies, or the adaption of existing methodology, to ensure that pragmatic, high-quality research is undertaken.

## Conclusions

Conducting trials in the police setting can be challenging and poses a number of methodological and practical challenges. However, it is important that RCTs are undertaken to ensure that policing initiatives are informed by a strong evidence base. Based on our experiences of the Connect trial we propose the following recommendations for the future conduct of trials in the police setting.

Collaborative approaches, such as co-production of evidence between police and academia, is needed to ensure that research addresses policing priorities and is rigorously conducted. In the Connect trial we ensured that evidence was co-produced in a number of ways. Firstly, we had police officers embedded within our Trial Management Group including police practitioners, senior police officers and data analysts. This is essential to ensure that those with detailed knowledge of the context in which research is being undertaken are involved in trial design. Secondly, police officers and data analysts were also co-authors on all trial papers and aided with the interpretation and reporting of trial findings. We also obtained feedback on lay summaries of trial findings that were then disseminated throughout the police force. Thirdly, we held ‘partners meetings’ with key stakeholders (e.g. police, charities, health service providers) throughout the trial period which provided an opportunity for the research team to obtain feedback on the trial design whilst also providing a forum for promoting and disseminating findings.

Given the complex nature of routinely collected police data, it is advised that future trials obtain engagement from police data analysts throughout the trial period. Police data analysts provided a unique insight into what data is recorded and its accuracy, and undertook data extraction.

The police routinely collect large amounts of rich data, which is currently stored on a number of different IT systems. Where possible, police forces may wish to consider re-designing their IT systems so that they are useful for research, analysis and operational purposes and to ensure that data collected is fit for purpose.

Future trials in the police setting may benefit from following the Medical Research Council’s Framework for designing and evaluating complex interventions to improve health [8]. The framework outlines the process of developing and evaluating complex interventions, through a series of phases which require significant pilot and feasibility work to not only develop and test the feasibility of the intervention but also key elements of trial design such as recruitment, sample size and outcomes. Adopting the MRC’s framework would be particularly useful for RCTs in the police setting as interventions are likely to be complex (i.e. made up of several interacting components) and are, as previously noted, likely to involve a number of challenges for trial design. Adopting the MRC’s framework would, therefore, enable researchers to conduct a series of pilot and feasibility studies to develop the intervention and address methodological issues relating to outcomes, recruitment, and sample sizes, before undertaking a full effectiveness trial.

Pragmatic trials should be adopted so that trial design can reflect the shifting landscape of policing and reflect the context to which findings will be applied. Additionally, it is acknowledged that there is often a need for research to be conducted quickly if it is to inform policy. However, it is important that sufficient lead-in time for trial set-up and obtaining necessary research governance approvals is considered when allocating and applying for research funding in this setting.

The Connect trial showed that undertaking an RCT of a complex intervention in the police setting is feasible within a short time frame, but lessons can be learnt. Future trials need to ensure that there is a reasonable amount of lead-in time before starting data collection to fully understand the policing context and what is possible within the project’s scope, whilst also taking into consideration the most appropriate length of follow-up time to show an effect. If service-user outcomes are to be collected, additional time may be required to obtain the necessary approvals.

## Abbreviations

Connect: Co-Production of Policing Evidence, Research and Training: focus mental health
CoP: College of Policing
EBP: Evidence Based Policing
MRC: Medical Research Council
NHS: National Health Service
RCT: Randomised controlled trial
SNC: Safer Neighbourhood Command
UK: United Kingdom
